# Associations of Long‐Term Blood Pressure Burden and Variability With Kidney Function Decline

**DOI:** 10.1111/jch.70306

**Published:** 2026-06-01

**Authors:** Sisi Xie, Peter Vollenweider, Julien Vaucher, Pedro Marques‐Vidal

**Affiliations:** ^1^ Department of Medicine, Internal Medicine Lausanne University Hospital (CHUV) and University of Lausanne Lausanne Switzerland; ^2^ Department of Medicine and Specialties, Internal Medicine Fribourg Hospital and University of Fribourg Fribourg Switzerland

**Keywords:** blood pressure variability, chronic kidney disease, cohort studies, cumulative blood pressure, eGFR slope, glomerular filtration rate

## Abstract

Long‐term blood pressure (BP) burden and visit‐to‐visit BP variability may better capture cumulative hemodynamic stress on the kidneys than single‐visit BP measurements, yet evidence linking these metrics with kidney function decline in the general population remains limited. We analyzed 2,821 adults with preserved kidney function from a prospective cohort study. Cumulative BP exposure and visit‐to‐visit BP variability were derived from BP measurements obtained at baseline and two follow‐up visits. Outcomes included annual estimated glomerular filtration rate (eGFR) slope and study‐defined incident chronic kidney disease (CKD). Higher long‐term BP exposure, particularly systolic BP (SBP), and greater BP variability were associated with faster kidney function decline. A 1‐standard deviation increase in cumulative SBP was associated with a more negative annual eGFR slope (β≈−0.15 to −0.18 mL/min/1.73 m^2^ per year). Cumulative SBP was also independently associated with study‐defined incident CKD (hazard ratio 1.45; 95% confidence interval 1.15–1.83), whereas associations for diastolic BP and BP variability were generally weaker. No evidence of nonlinearity or significant effect modification was observed. These findings suggest that long‐term BP burden, particularly cumulative SBP, is associated with accelerated kidney function decline in the general population.

## 1 Introduction

Chronic kidney disease (CKD) has become a major global public health concern, with its prevalence and mortality rising steadily over recent decades. By 2040, CKD is projected to rank as the fifth leading cause of years of life lost worldwide [1]. Hypertension is one of the most important modifiable risk factors for the development and progression of CKD [2]. However, most studies assessing blood pressure (BP)‐related kidney risk have relied on single BP measurements, which may not adequately capture an individual's long‐term hemodynamic burden or its dynamic fluctuations [3, 4, 5].

Because hypertension‐induced kidney injury is driven by cumulative and sustained hemodynamic load, cumulative BP exposure may better reflect the chronic pressure stress on the kidneys than BP measured at a single visit. Single‐visit BP measurements provide only a snapshot of an individual's blood pressure and may be influenced by short‐term fluctuations, measurement error, or transient conditions. As a result, they may not adequately capture long‐term exposure to elevated BP or the dynamic patterns of BP over time. In contrast, cumulative BP integrates both the intensity and duration of BP exposure, while BP variability reflects fluctuations in BP that may impose additional hemodynamic stress on the kidney microvasculature. Recent studies have highlighted cumulative BP burden and BP variability as markers of vascular stress and predictors of adverse cardiovascular outcomes [6, 7, 8, 9]. Yet, evidence linking long‐term BP exposure and BP variability with kidney function decline remains limited.

Cumulative BP exposure has been increasingly recognized as a marker of long‐term hemodynamic burden and has been linked to adverse kidney outcomes in several cohort studies. Two recent cohort studies from Asian populations reported that higher cumulative BP burden was associated with increased risk of diabetic kidney disease progression or incident CKD [10, 11]. Similarly, cumulative systolic BP exposure across the life course, from childhood or young adulthood to midlife, has been associated with an increased risk of albuminuria in midlife, suggesting that prolonged BP load contributes to early kidney microvascular injury [12, 13]. Another study among hypertensive patients found that BP load derived from ambulatory BP monitoring was associated with accelerated kidney function decline [14].

In parallel, visit‐to‐visit BP variability has emerged as a complementary indicator of unstable BP control. Evidence from large clinical trials such as ALLHAT and other observational studies has shown that greater BP variability is associated with adverse CKD outcomes [15, 16, 17]. However, findings have not been entirely consistent; in ONTARGET and TRANSCEND, visit‐to‐visit systolic BP variability showed limited independent predictive value for renal outcomes compared with mean on‐treatment systolic blood pressure [18]. These mixed findings suggest that the role of BP variability in kidney disease progression remains uncertain.

From a mechanistic perspective, cumulative BP exposure and BP variability may contribute to kidney damage through distinct but complementary pathways. Chronic exposure to elevated BP leads to sustained glomerular hypertension and progressive structural kidney damage, whereas increased BP variability may reflect impaired vascular regulation and promote intermittent hemodynamic stress, endothelial dysfunction, oxidative stress, and microvascular injury [19, 20, 21, 22].

However, these studies were largely restricted to high‐risk or disease‐specific populations, relied on threshold‐based BP load metrics, focused primarily on albuminuria or categorical kidney outcomes, and rarely evaluated multiple complementary BP exposure indicators or continuous measures of kidney function decline. Evidence from general adult populations, particularly in European settings, remains relatively limited. This gap is important because most existing evidence has been derived from Asian or high‐risk populations, which may differ from European populations in terms of blood pressure distribution, cardiovascular risk profiles, healthcare systems, and CKD epidemiology. Therefore, the generalizability of prior findings to European populations remains uncertain, underscoring the need for population‐based studies in European settings.

To address these gaps, we examined the associations of several BP exposure metrics, including cumulative BP, time‐weighted average BP, and visit‐to‐visit BP variability, with the annual estimated glomerular filtration rate (eGFR) slope and incident CKD in a population‐based prospective cohort from Switzerland. We hypothesized that higher cumulative BP exposure and greater BP variability would be associated with a faster decline in eGFR and a higher risk of developing CKD.

## 2 Participants and Methods

### 2.1 Study Population

The CoLaus study is an ongoing population‐based cohort initiated in 2003 to investigate the epidemiology and genetic determinants of cardiovascular and metabolic disorders [23]. The baseline examination was conducted between 2003 and 2006 and included 6733 participants aged 35–75 years. Three subsequent follow‐up surveys were performed in 2009–2012 (follow‐up 1, FU1), 2014–2017 (follow‐up 2, FU2), and 2018–2021 (follow‐up 3, FU3), respectively, during which detailed clinical, biochemical, and lifestyle data were repeatedly collected.

For the present analysis, blood pressure exposure was assessed using BP measurements collected from baseline through FU2, while kidney outcomes were evaluated using eGFR measurements obtained between FU2 and FU3.

### 2.2 Blood Pressure Assessment and Exposure Metrics

BP was assessed at three visits: baseline (*t*_0_), follow‐up 1 (*t*_1_), and follow‐up 2 (*t*_2_). At each visit, trained staff measured systolic blood pressure (SBP) and diastolic blood pressure (DBP) using an Omron HEM‐907 automated oscillometric sphygmomanometer after participants rested for at least 10 min in a seated position. The mean of the last two consecutive readings was recorded.

BP measurements from baseline through FU2 were used to derive long‐term exposure metrics. Cumulative BP exposure was calculated using the trapezoidal method to approximate the area under the BP–time curve. For participants with measurements at both *t*_1_ and *t*_2_, cumulative BP was computed as:

(1) $$B\;P_{cum}=\left(\frac{BP_{0}+BP_{1}}{2}\right)\;\left(t_{1}-t_{0}\right)+\left(\frac{BP_{1}+BP_{2}}{2}\right)\left(t_{2}-t_{1}\right)$$

If a participant did not attend FU1, cumulative BP was calculated using only baseline and FU2 values:

(2) $$B\;P_{cum}=\left(\frac{BP_{0}+BP_{2}}{2}\right)\;\left(t_{2}-t_{0}\right)$$

Cumulative BP reflects the overall BP burden and corresponds to the integral of BP exposure over time. Time‐weighted average BP was defined as cumulative BP divided by the total duration of follow‐up from baseline to FU2:

(3) $$B\;P_{twa}=\frac{BP_{cum}}{t_{2}-t_{0}}$$

where *BP*_cum_ denotes cumulative BP burden, and *BP*_twa_ denotes time‐weighted average BP.

Visit‐to‐visit BP variability across *t*_0_, *t*_1_, and *t*_2_ was assessed using three established indices: standard deviation (SD), coefficient of variation (CV), and variability independent of the mean (VIM). These indices were selected because they capture complementary aspects of BP variability. SD reflects the absolute dispersion of BP measurements, CV standardizes variability relative to the mean BP level, and VIM is designed to assess variability independent of mean BP, thereby reducing the potential influence of average BP levels on variability estimates. These measures provide a more comprehensive characterization of visit‐to‐visit BP variability. Only participants who attended FU2 were included in variability calculations.

(4) $$SD=\sqrt{\frac{\sum {\left(BP_{i}-\bar{BP}\right)}^{2}}{n-1}}$$

(5) $$CV\;\left(\%\right)=\frac{SD}{\bar{BP}}\;\times 100$$

(6) $$VIM=SD\times {\left(\frac{BP_{overall}}{\bar{BP}}\right)}^{k}$$

where BP overall represents the overall mean BP of the study population. The exponent k was estimated from the regression:

(7) $$\ln\left(SD\right)\;=\;\alpha +k\;\ln\left(\bar{BP}\right)$$

**FIGURE 1:**
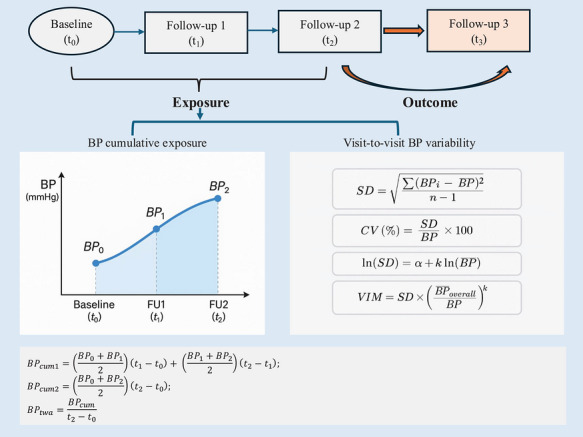
Study design and blood pressure exposure metrics. Cumulative BP exposure was calculated using BP measurements at baseline (*t*_0_), follow‐up 1 (*t*_1_), and follow‐up 2 (*t*_2_). When both *t*_1_ and *t*_2_ measurements were available, cumulative exposure was computed as the sum of the interval‐specific mean BP multiplied by the corresponding time duration. When only the baseline and *t*_2_ measurements were available, cumulative BP was calculated over the entire baseline–*t*_2_ interval. Time‐weighted average BP was defined as cumulative BP divided by total follow‐up time. BP variability was assessed using SD, CV, and VIM, reflecting absolute, relative, and mean‐independent variability, respectively. The BP trajectories and calculations shown are schematic illustrations of the exposure definitions.

All BP exposure metrics (cumulative BP, time‐weighted average BP, SD, CV, VIM) were standardized to 1 standard deviation before inclusion in regression models. This standardization allows direct comparison of effect estimates across different BP metrics with varying units and scales, and expresses associations per 1‐standard deviation increase, thereby improving interpretability. The corresponding means and standard deviations of the BP exposure metrics are provided in Table S1. An overview of the study timeline and BP metric derivation is shown in Figure 1.

### 2.3 Kidney Outcomes

The primary outcome was the annual eGFR slope between FU2 and FU3, reflecting the rate of kidney function decline. Serum creatinine was measured using an enzymatic assay calibrated to an isotope dilution mass spectrometry, and the mean of repeated internal measurements was used. eGFR was calculated using the 2021 Chronic Kidney Disease Epidemiology Collaboration (CKD‐EPI) creatinine‐based equation [24].

The annual eGFR slope was computed as:

$$\mathrm{eGFR}\;\mathrm{slope}\;=\frac{eGFR_{FU3}-eGFR_{FU2}}{\left(\mathrm{days}\;\mathrm{between}\;\mathrm{FU}2\;\mathrm{and}\;\mathrm{FU}3\right)/365.25}$$

More negative values indicate faster kidney function decline.

The secondary outcome was study‐defined incident CKD, defined as an eGFR <60 mL/min/1.73 m^2^ at FU3. Because eGFR was measured only at scheduled survey visits, persistence of reduced eGFR for at least 3 months could not be confirmed according to Kidney Disease: Improving Global Outcomes (KDIGO) criteria. Therefore, this outcome should be interpreted as study‐defined CKD rather than clinically confirmed CKD [25]. This limitation is common in longitudinal population‐based cohort studies with periodic follow‐up assessments. For readability, the term “incident CKD” is used throughout the manuscript to refer to this study‐defined outcome based on scheduled eGFR measurements. Albuminuria (assessed as microalbuminuria) was not included because of limited availability during follow‐up.

### 2.4 Covariates

Covariates included age (years), sex (male/female), education level (low/middle/high), smoking status (never/former/current), weekly alcohol consumption (units/week), diabetes mellitus (yes/no), body mass index (BMI, kg/m^2^), and low‐density lipoprotein (LDL) cholesterol (mmol/L).

Education was categorized into high (university), middle (high school), and low (apprenticeship or mandatory schooling). Usual alcohol consumption was self‐reported and expressed as the number of units (glasses of wine, bottles or cans of beer, or shots of spirits) consumed per week. Smoking status was self‐reported and classified as never, former (regardless of time since cessation), or current. Body weight and height were measured with participants barefoot and wearing light indoor clothing using calibrated Seca devices. BMI was calculated as weight (kg) divided by height (m) squared. Fasting plasma glucose was determined using a glucose dehydrogenase method. Diabetes mellitus was defined as fasting plasma glucose ≥7.0 mmol/L or the use of oral hypoglycemic medications or insulin therapy.

### 2.5 Inclusion and Exclusion Criteria

Participants enrolled at baseline were eligible for inclusion. We then excluded individuals who met the following criteria: (1) eGFR <60 mL/min/1.73 m^2^ at FU2 (to exclude prevalent CKD), (2) did not attend the FU2 or FU3 follow‐up visit, (3) had missing BP measurements required to compute cumulative BP exposure, and (4) had missing data on any covariates included in the adjusted models. Participants with missing data on BP exposure variables or covariates were excluded from the analysis; therefore, a complete‐case approach was used for all analyses.

### 2.6 Statistical Analysis

Statistical analyses were conducted using Stata v.18 (Stata Corp, College Station, TX, USA) and RStudio Desktop (Version 2024.04.2+764). Continuous variables are presented as means ± SD, and categorical variables as counts and percentages. Differences across groups were assessed using Student's *t*‐tests or *χ* ^2^ tests as appropriate. All BP exposure metrics, including cumulative BP, time‐weighted average BP, and visit‐to‐visit BP variability indices (SD, CV, and VIM), were standardized to 1 SD before analysis.

The associations of blood pressure exposure metrics with the annual eGFR slope were evaluated using multivariable linear regression. The associations between BP metrics and incident CKD were examined using Cox proportional hazards models, and results are reported as hazard ratios (HRs) with 95% confidence intervals (CIs). For Cox proportional hazards analyses, follow‐up time was defined as the interval between the FU2 and FU3 examinations. Participants contributed person‐time from FU2 until the FU3 visit, at which study‐defined incident CKD status was assessed. Because kidney outcomes were assessed only at scheduled study visits, the exact timing of CKD onset between study visits was not available. Cox proportional hazards models were used to account for differences in follow‐up duration across participants, and hazard ratios should therefore be interpreted as relative risk estimates over the follow‐up interval rather than precise estimates of instantaneous event risk.

Three multivariable models were fitted for both outcomes. Model 1 was adjusted for age, sex, education level, smoking status, alcohol consumption, body mass index, diabetes, LDL cholesterol, and antihypertensive medication use at FU2. Model 2 included all covariates in Model 1 with additional adjustment for baseline eGFR. Model 3 included all covariates in Model 1 with additional adjustment for eGFR at FU2. Models 2 and 3 adjusted for kidney function measured at baseline or at FU2, respectively, to assess whether associations were sensitive to the timing of kidney function assessment before CKD follow‐up; these models were not intended as sequentially nested causal models.

Restricted cubic spline analyses were performed using Model 1 covariate adjustment to evaluate potential non‐linear associations between cumulative SBP and kidney outcomes. Linear regression models with Model 1 covariate adjustment were used to test for linear trends across quartiles of BP variability. Subgroup analyses were conducted to assess potential effect modification by age, sex, BMI, smoking status, hypertension status, diabetes, and antihypertensive medication use; interactions were tested by including cross‐product terms between cumulative SBP and each stratifying variable.

A sensitivity analysis was conducted in which primary models were repeated using the percent annual change in eGFR as an alternative measure of kidney function decline. To account for multiple comparisons across blood pressure exposure metrics, false discovery rate (FDR) adjusted q values were calculated using the Benjamini–Hochberg method for Model 3 estimates. All statistical tests were two‐sided, and *p*‐values <0.05 were considered statistically significant.

## 3 Results

### 3.1 Participant Characteristics

From the original cohort, we excluded participants with: (1) eGFR <60 mL/min/1.73 m^2^ at FU2 (*n =* 300); (2) no attendance at the FU2 or FU3 follow‐up visit (*n =* 3156); (3) missing BP measurements required to compute cumulative BP exposure (*n =* 14); and (4) missing data on any covariates included in the adjusted models (*n =* 442). A total of 2821 participants were included in the final analyses (Figure S1).

Among these participants (mean age 60.6 years, 55% women), the mean baseline eGFR was 91.1 mL/min/1.73 m^2^, and the mean eGFR at FU2 was 87.3 mL/min/1.73 m^2^. The characteristics of participants according to incident CKD status are presented in Table 1. Compared with those without incident CKD, participants who developed CKD were older, had lower educational attainment, were more likely to have diabetes, and had higher BMI and SBP at FU2. They also had lower baseline and FU2 eGFR and were more likely to use antihypertensive medications (Table 1). Characteristics of included and excluded participants at study baseline and FU2 are presented in Tables S2 and S3. Compared with included participants, excluded participants were generally older and had a less favorable cardiovascular risk profile, including higher systolic and diastolic blood pressure, higher BMI, and a greater prevalence of diabetes. In contrast, kidney function at FU2 was broadly similar between groups.

**TABLE 1:** Characteristics of participants at follow‐up 2, stratified by incident CKD between follow‐up 2 and follow‐up 3.

Variables	Overall (*n =* 2821)	No incident CKD (*n =* 2720)	Incident CKD (*n =* 101)	*p*‐value
Age, years	60.6 ± 9.3	60.2 ± 9.2	70.4 ± 8.2	**<0.001**
Female sex, n(%)	1555 (55.1)	1500 (55.2)	55 (54.5)	0.891
Education level, n(%)				**0.039**
Low	1309 (46.4)	1256 (46.2)	53 (52.5)	
Middle	819 (29.0)	785 (28.8)	34 (33.6)	
High	693 (24.6)	679 (25.0)	14 (13.9)	
Smoking status, n(%)				0.094
Never	1212 (43.0)	1158 (42.6)	54 (53.5)	
Former	1104 (39.1)	1072 (39.4)	32 (31.7)	
Current	505 (17.9)	490 (18.0)	15 (14.8)	
Alcohol consumption(unit), n(%)				0.202
None	659 (23.3)	629 (23.1)	30 (29.7)	
1–13/week	1771 (62.8)	1711 (62.9)	60 (59.4)	
14–27/week	321 (11.4)	310 (11.4)	11 (10.9)	
28+/week	70 (2.5)	70 (2.6)	0 (0)	
BMI, kg/m^2^	26.0 ± 4.5	26.0 ± 4.5	27.1 ± 4.4	**0.017**
SBP, mmHg	124.7 ± 16.6	124.3 ± 16.4	133.9 ± 19.3	**<0.001**
DBP, mmHg	77.1 ± 10.2	77.1 ± 10.1	76.8 ± 11	0.747
Diabetes, n(%)	192 (6.8)	175 (6.4)	17 (16.8)	**<0.001**
LDL cholesterol, mmol/L	3.2 ± 0.9	3.2 ± 0.9	3.1 ± 1.1	0.102
Antihypertensive medication, n(%)	750 (26.6)	695 (25.6)	55 (54.5)	**<0.001**
Baseline eGFR, mL/min/1.73m^2^	91.1 ± 13.7	91.5 ± 13.5	79.1 ± 12.3	**<0.001**
FU2 eGFR, mL/min/1.73m^2^	87.3 ± 12.5	88.0 ± 12.1	69.2 ± 8.1	**<0.001**

*Note*: Results are expressed as the number of participants (column percentage) for categorical variables and as mean ± SD for continuous variables. Between‐group comparisons were performed using chi‐square for categorical variables and Student's t‐test. Bold *p*‐values indicate *p* < 0.05.

### 3.2 Associations of BP Exposure Metrics with Annual eGFR Decline

All blood pressure exposure metrics were significantly associated with a faster annual decline in eGFR (Table 2). Higher cumulative SBP showed the strongest association, with a 1‐SD increase corresponding to an additional decline of approximately 0.15–0.18 mL/min/1.73 m^2^ per year across the three models. Similar associations were observed for time‐weighted average SBP and for SBP variability indices (SD, CV, and VIM), all of which were consistently linked to a more negative eGFR slope. Associations for DBP exposure and variability metrics were directionally similar but of smaller magnitude. Among variability metrics, SBP_SD demonstrated the strongest association (*β* = −0.15; 95% CI, −0.24 to −0.06).

**TABLE 2:** Associations of blood pressure metrics (per 1‐SD increase) with the annual eGFR slope between follow‐up 2 and follow‐up 3 across three adjustment models.

BP metric (per 1‐SD)	Model 1 β (95%CI)	*p*‐value	Model 2 β (95%CI)	*p*‐value	Model 3 β (95%CI)	*p*‐value	FDR (q)
SBP_cum_	−0.18 (−0.29, −0.07)	**0.001**	−0.18 (−0.29, −0.07)	**0.001**	−0.15 (−0.26, −0.05)	**0.003**	**0.005**
SBP_twa_	−0.17 (−0.28, −0.06)	**0.002**	−0.17 (−0.28, −0.06)	**0.003**	−0.15 (−0.25, −0.04)	**0.006**	**0.009**
SBP_SD	−0.15 (−0.24, −0.06)	**0.001**	−0.15 (−0.24, −0.06)	**0.001**	−0.15 (−0.24, −0.06)	**0.001**	**0.005**
SBP_CV	−0.13 (−0.22, −0.04)	**0.004**	−0.13 (−0.22, −0.04)	**0.004**	−0.14 (−0.22, −0.05)	**0.002**	**0.005**
SBP_VIM	−0.11 (−0.19, −0.02)	**0.019**	−0.11 (−0.19, −0.02)	**0.019**	−0.11 (−0.20, −0.03)	**0.008**	**0.010**
DBP_cum_	−0.13 (−0.23, −0.03)	**0.009**	−0.13 (−0.22, −0.03)	**0.011**	−0.12 (−0.21, −0.03)	**0.011**	**0.012**
DBP_twa_	−0.12 (−0.21, −0.02)	**0.020**	−0.11 (−0.21, −0.01)	**0.026**	−0.11 (−0.20, −0.01)	**0.024**	**0.024**
DBP_SD	−0.15 (−0.24, −0.06)	**0.001**	−0.15 (−0.24, −0.06)	**0.002**	−0.15 (−0.23, −0.06)	**0.001**	**0.005**
DBP_CV	−0.13 (−0.22, −0.04)	**0.003**	−0.13 (−0.22, −0.04)	**0.003**	−0.13 (−0.22, −0.05)	**0.002**	**0.005**
DBP_VIM	−0.13 (−0.22, −0.04)	**0.004**	−0.13 (−0.22, −0.04)	**0.004**	−0.13 (−0.22, −0.05)	**0.002**	**0.005**

*Note*: Results are expressed as adjusted β coefficients and 95% CIs derived from multivariable linear regression models. Blood pressure metrics were standardized (per 1‐SD increase). Model 1 was adjusted for age, sex, education, smoking status, alcohol consumption, body mass index, diabetes, LDL cholesterol, and antihypertensive medication use at FU2. Model 2 included all covariates in Model 1 with additional adjustment for baseline eGFR. Model 3 included all covariates in Model 1 with additional adjustment for eGFR at FU2; FDR‐adjusted q values were calculated using the Benjamini–Hochberg method. The annual eGFR slope was calculated as the difference between eGFR at FU3 and FU2 divided by the exact follow‐up time between FU2 and FU3 (days/365.25). Bold *p*‐values indicate *p* < 0.05, and bold FDR(q) values indicate q < 0.05.

**FIGURE 2:**
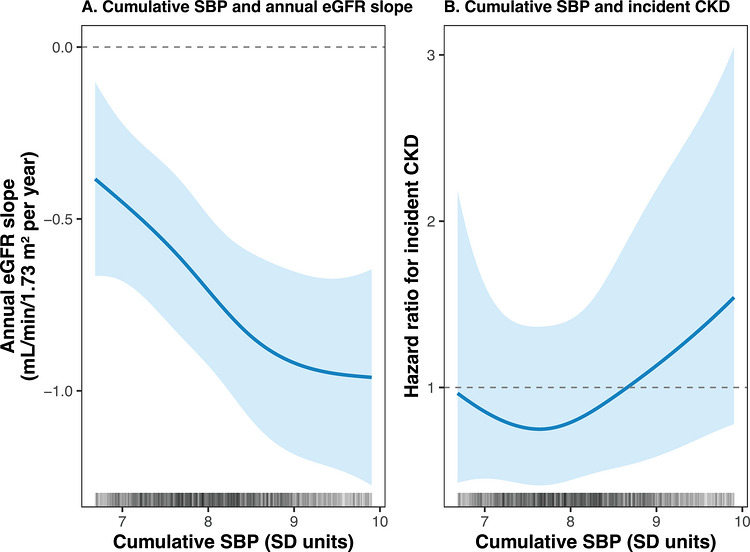
Restricted cubic splines showing the associations of cumulative systolic blood pressure with annual eGFR slope and incident CKD between follow‐up 2 and follow‐up 3. Restricted cubic splines with 4 knots were used to model the associations between SBP_cum_ (per SD unit), the annual eGFR slope, and incident CKD. The solid line represents the adjusted effect estimate and the shaded area the 95% confidence interval. No evidence of nonlinearity was detected (P_nonlin = 0.415 for eGFR slope; 0.389 for CKD). Cumulative SBP was modeled per 1 SD (SD = 163.6 mmHg·year). Models were adjusted for age, sex, education, smoking status, alcohol consumption, body mass index, diabetes, LDL cholesterol, and antihypertensive medication use at FU2.

Restricted cubic spline analyses further supported an approximately linear association between cumulative SBP and annual eGFR decline, with no evidence of a threshold effect or nonlinearity (*p* for nonlinearity = 0.415; Figure 2).

Overall, these associations were directionally consistent across all models, and all associations between BP exposure metrics and annual eGFR slope remained statistically significant after FDR correction (Table 2).

### 3.3 Associations of BP Exposure Metrics with Incident CKD

Higher cumulative SBP was significantly associated with an increased risk of incident CKD (Table 3). In model 3, which additionally adjusted for FU2 eGFR, the association between cumulative SBP and incident CKD remained significant and slightly strengthened (HR = 1.45; 95% CI, 1.15–1.83). Time‐weighted average SBP and SBP_SD also showed a nominal association in model 3, although the effect sizes were smaller. Other SBP variability metrics (CV and VIM) demonstrated positive but non‐significant associations with CKD risk.

**TABLE 3:** Associations of blood pressure metrics (per 1‐SD increase) with incident CKD between follow‐up 2 and follow‐up 3.

BP metric (per 1‐SD)	Model 1 Incident CKD HR (95%CI)	*p*‐value	Model 2 Incident CKD HR (95%CI)	*p*‐value	Model 3 Incident CKD HR (95%CI)	*p*‐value	FDR (q)
SBP_cum_	1.31 (1.04, 1.64)	**0.020**	1.28 (1.03, 1.60)	**0.028**	1.45 (1.15, 1.83)	**0.002**	**0.020**
SBP_twa_	1.17 (0.94, 1.47)	0.164	1.15 (0.92, 1.44)	0.215	1.30 (1.03, 1.64)	**0.026**	0.088
SBP_SD	1.10 (0.94, 1.29)	0.227	1.14 (0.97, 1.34)	0.121	1.20 (1.01, 1.43)	**0.043**	0.108
SBP_CV	1.08 (0.92, 1.28)	0.357	1.12 (0.95, 1.33)	0.183	1.15 (0.96, 1.39)	0.126	0.210
SBP_VIM	1.05 (0.88, 1.25)	0.591	1.10 (0.91, 1.32)	0.332	1.09 (0.90, 1.32)	0.386	0.485
DBP_cum_	1.20 (0.96, 1.50)	0.112	1.15 (0.91, 1.45)	0.234	1.33 (1.05, 1.68)	**0.019**	0.088
DBP_twa_	1.08 (0.86, 1.34)	0.511	1.02 (0.82, 1.28)	0.839	1.20 (0.95, 1.50)	0.122	0.210
DBP_SD	1.08 (0.90, 1.29)	0.391	1.08 (0.91, 1.29)	0.372	1.08 (0.90, 1.30)	0.388	0.485
DBP_CV	1.07 (0.90, 1.28)	0.436	1.09 (0.91, 1.29)	0.351	1.06 (0.88, 1.27)	0.532	0.548
DBP_VIM	1.07 (0.90, 1.28)	0.442	1.09 (0.91, 1.29)	0.350	1.06 (0.88, 1.27)	0.548	0.548

*Note*: Results are expressed as adjusted HRs and 95% CIs from multivariable Cox proportional hazards models. Blood pressure metrics were standardized (per 1‐SD increase). Model 1 was adjusted for age, sex, education, smoking status, alcohol consumption, body mass index, diabetes, LDL cholesterol, and antihypertensive medication use at FU2. Model 2 included all covariates in Model 1 with additional adjustment for baseline eGFR. Model 3 included all covariates in Model 1 with additional adjustment for eGFR at FU2. FDR‐adjusted q values were calculated using the Benjamini–Hochberg method. Bold *p*‐values indicate *p* < 0.05, and bold FDR(q) values indicate q < 0.05.

For DBP exposure measures, most associations were modest and not statistically significant, except for cumulative DBP, which was associated with a 33% higher risk of CKD in model 3 (HR = 1.33; 95% CI, 1.05–1.68).

Restricted cubic spline analyses similarly supported an approximately linear association between cumulative SBP and incident CKD, with no evidence of nonlinearity or a distinct threshold effect (*p* for nonlinearity = 0.389; Figure 2).

Overall, systolic BP measures, particularly cumulative exposure, showed stronger and more consistent associations with incident CKD than diastolic measures. After FDR correction, only the association for cumulative SBP remained statistically significant (Table 3).

### 3.4 Systolic and Diastolic BP Variability and Annual eGFR Decline

**FIGURE 3:**
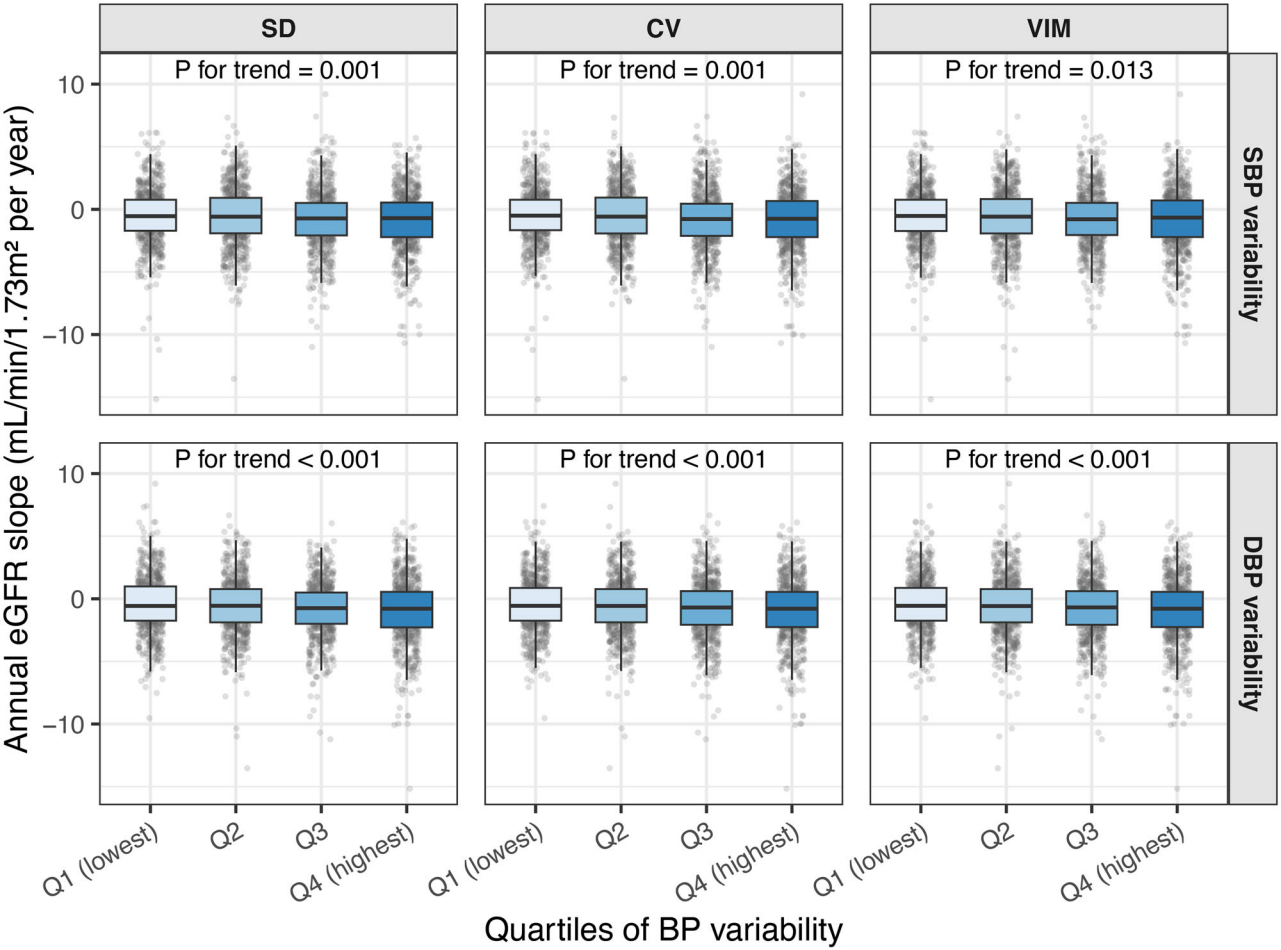
Associations of blood pressure variability quartiles with annual eGFR slope between follow‐up 2 and follow‐up 3. Boxplots show the annual eGFR slope across quartiles of systolic (top row) and diastolic (bottom row) blood pressure variability, including SD, CV, and VIM. Q1 represents the lowest variability and Q4 the highest. A more negative eGFR slope indicates a faster decline in kidney function. *p*‐values for trend were derived from linear regression models adjusting for age, sex, education level, smoking status, alcohol consumption, body mass index, diabetes, LDL cholesterol, and antihypertensive medication use at FU2.

Higher visit‐to‐visit BP variability was consistently associated with a faster annual decline in eGFR. As shown in Figure 3, participants in the highest quartile of SBP variability (SD, CV, or VIM) exhibited the fastest decline of kidney function, whereas those in the lowest quartile showed the slowest decline. Trend tests from linear regression were significant for all SBP variability metrics.

DBP variability showed similar patterns, with DBP_SD, DBP_CV, and DBP_VIM being significantly associated with a steeper decline in eGFR. Overall, these results indicate that greater visit‐to‐visit BP variability is an important predictor of accelerated kidney function loss, with SBP variability showing slightly stronger associations than DBP variability.

### 3.5 Subgroup Analyses

Subgroup analyses showed that the association between cumulative SBP and annual eGFR decline was broadly similar across participant characteristics, including age, sex, BMI, smoking status, diabetes, hypertension status, and antihypertensive medication use (Figure S2). Although the magnitude of association varied slightly among subgroups, none of the interaction tests reached statistical significance (all *p* for interaction>0.05), indicating no evidence of effect modification.

### 3.6 Sensitivity Analyses Using Annual Percent Change in eGFR

Sensitivity analyses using the annual percent change in eGFR produced results consistent with the primary analyses (Table S4). Higher cumulative SBP, time‐weighted average SBP, and SBP variability metrics (SD, CV, and VIM) remained significantly associated with faster kidney function decline, with effect estimates comparable in magnitude and direction to those observed for the absolute annual eGFR slope. DBP exposure and DBP variability metrics showed similar but weaker associations. These findings support the robustness of the primary results.

## 4 Discussion

### 4.1 Principal Findings

In this prospective cohort of adults free of CKD at FU2, higher long‐term BP exposure and greater visit‐to‐visit BP variability were consistently associated with faster kidney function decline. Cumulative SBP showed the strongest association with a more negative annual eGFR slope, and this relationship remained robust across all adjustment models. Cumulative SBP was also independently associated with a higher incidence of CKD, with systolic measures demonstrating stronger and more consistent associations than diastolic measures. These findings were generally consistent across subgroups and were confirmed in sensitivity analyses using the annual percent change in eGFR as an alternative outcome.

### 4.2 Comparison with Previous Studies

Our findings are consistent with prior studies conducted in Asian populations, in which higher cumulative BP burden was associated with a greater risk of kidney impairment. Building on this, an increasing number of cohort studies have adopted cumulative or trajectory‐based BP metrics in recent years. For example, prospective studies from Korea and China have demonstrated that higher cumulative systolic and diastolic BP loads during follow‐up were positively associated with incident CKD or diabetic kidney disease, suggesting that among individuals with similar BP control at single time points, the long‐term burden of BP exposure may be a key determinant of kidney outcomes [10, 11]. Similarly, a study in hypertensive Han Chinese patients further showed that cumulative BP load derived from 24‐hour ambulatory BP monitoring was closely associated with long‐term kidney function decline, indicating that clinic BP readings alone may underestimate the true hemodynamic burden [14].

In addition, trajectory‐based analyses have shown that individuals with persistently high or progressively increasing BP trajectories have substantially higher risks of incident CKD or rapid kidney function decline compared with those maintaining stable BP levels, whereas individuals with decreasing or consistently low trajectories tend to have more favorable outcomes [26, 27]. These findings further support that the cumulative effect of “exposure duration × exposure intensity” provides superior prognostic information compared with single BP measurements.

Long‐term follow‐up studies from childhood to adulthood have also demonstrated that higher cumulative systolic BP exposure is significantly associated with elevated midlife urine albumin‐to‐creatinine ratio and subclinical kidney damage. These associations remained robust after adjustment for multiple confounders, suggesting that sustained BP load beginning early in life may initiate glomerular and microvascular injury at a relatively young age [12]. Likewise, subsequent work has shown that long‐term visit‐to‐visit BP variability is independently associated with subclinical kidney damage and albuminuria in midlife, indicating that not only cumulative BP burden but also greater BP fluctuation may contribute to kidney injury [28].

Moreover, the age of hypertension onset also appears to be relevant to adult kidney health. A recent analysis of U.S. NHANES data found that individuals with earlier‐onset hypertension had a higher prevalence of CKD, and this association remained generally consistent across sex and diabetes subgroups. These findings suggest that earlier onset may reflect longer cumulative BP exposure, which is linked to increased kidney risk [29].

Despite these consistent findings, not all BP metrics were significantly associated with kidney outcomes in this study. Some SBP variability indices (such as CV and VIM) and most diastolic BP measures showed weaker or non‐significant associations with incident CKD. Several factors may explain these findings. First, systolic BP may play a more prominent role in arterial stiffness and microvascular damage, which are key mechanisms underlying kidney function decline, whereas diastolic BP may have a more limited contribution in this context. Second, variability measures derived from a limited number of visits may be subject to measurement error and may not fully capture long‐term BP fluctuations. Finally, differences in statistical power and effect size may have contributed to the lack of statistically significant associations for some metrics.

Taken together, despite some weaker or non‐significant associations observed for certain BP metrics, our results align closely with epidemiological evidence from Asian and other populations: whether assessed through cumulative BP load, longitudinal BP trajectories, or age of hypertension onset, greater long‐term BP exposure is associated with a higher risk of kidney impairment and incident CKD.

### 4.3 Potential Biological Mechanisms

Multiple biological mechanisms may account for the associations between long‐term BP exposure, BP variability, and subsequent kidney function decline. Sustained elevations in BP increase mechanical stress on the kidney microvasculature, leading to afferent arteriolar hyalinosis, impaired autoregulatory capacity, chronic glomerular hypertension, and progressive glomerulosclerosis and interstitial fibrosis [19, 20]. As autoregulation deteriorates, the kidney becomes increasingly sensitive to systemic BP fluctuations, thereby accelerating the decline in glomerular filtration rate [30, 31, 32]. Beyond cumulative burden alone, BP variability may exert additional deleterious effects through intermittent shear stress, endothelial dysfunction, microvascular injury, oxidative stress, and activation of inflammatory and fibrotic pathways [21, 22]. These hemodynamic oscillations may further disrupt autoregulation, amplify glomerular pressure swings, and induce episodic ischemia–reperfusion injury [33].

Beyond these hemodynamic effects, several molecular pathways may further contribute to hypertensive kidney injury. Chronic activation of the renin–angiotensin–aldosterone system (RAAS) plays a central role in hypertensive renal damage. Angiotensin II promotes renal vasoconstriction and intraglomerular hypertension and contributes to oxidative stress through increased production of reactive oxygen species via NADPH oxidase and mitochondrial pathways [34]. It can also activate angiotensin receptor and nuclear factor kappa‐light‐chain‐enhancer of activated B cells‐dependent inflammatory signaling, thereby promoting renal inflammation [35].

In addition to RAAS‐mediated effects, hypertension is associated with systemic oxidative stress, reduced nitric oxide bioavailability, and endothelial dysfunction, which may contribute to microvascular injury and impaired renal autoregulation [36]. In parallel, activation of profibrotic pathways, particularly the transforming growth factor‐β/Smad signaling cascade, promotes extracellular matrix accumulation and tubulointerstitial fibrosis [37].

Emerging evidence further implicates innate immune mechanisms, including inflammasome activation, in the amplification of renal inflammation and fibrosis, although this pathway is less well established in hypertensive kidney injury specifically [38, 39].

Together, these hemodynamic, oxidative, inflammatory, and profibrotic processes may interact under sustained blood pressure elevation, leading to progressive and potentially irreversible kidney damage. This provides a biologically plausible explanation for the association between cumulative systolic blood pressure exposure and kidney function decline observed in our study.

### 4.4 Practical Applications

Long‐term cumulative blood pressure exposure, particularly cumulative systolic blood pressure, provides prognostic information beyond single‐visit blood pressure measurements for kidney function decline. Assessment of visit‐to‐visit blood pressure variability may help identify individuals at increased risk of accelerated kidney function decline, even in the absence of baseline chronic kidney disease. The approximately linear associations observed in the spline analyses suggest that kidney risk may increase progressively across the range of cumulative SBP exposure, rather than only above a specific threshold. Clinically, this may indicate that even modest long‐term elevations in systolic blood pressure could contribute to kidney function decline, reinforcing the importance of sustained BP control across the full BP distribution rather than only above conventional hypertension thresholds.

From a practical perspective, these findings suggest that reliance on single‐visit BP measurements may underestimate long‐term risk. Incorporating repeated BP assessments over time and evaluating cumulative BP exposure may improve risk stratification for kidney function decline. In addition, monitoring visit‐to‐visit BP variability may help identify individuals with unstable BP control who could benefit from closer follow‐up or more intensive BP management. Emerging wearable BP monitoring technologies may further facilitate frequent and longitudinal BP assessment with minimal patient burden, potentially enabling improved identification of BP variability patterns and high‐risk clinical phenotypes [40].

### 4.5 Strengths and Limitations

This study has several notable strengths. First, it was based on a large, well‐characterized European population cohort, allowing evaluation of long‐term BP exposure in a general adult population rather than in high‐risk or disease‐specific groups. Second, repeated BP assessments over more than a decade enabled the derivation of multiple complementary BP exposure metrics, including cumulative BP, time‐weighted average BP, and several indices of visit‐to‐visit BP variability. Third, we assessed kidney outcomes using both a continuous measure of kidney function decline (annual eGFR slope) and incident CKD, allowing sensitive detection of early functional changes with clear clinical relevance.

Several limitations should be acknowledged. First, as an observational study, causal inference cannot be established. Second, BP was assessed during clinic visits rather than by ambulatory or home monitoring, which may not fully capture short‐term BP variability. Third, CKD outcomes were defined based on eGFR measurements, as albuminuria data were not consistently available during follow‐up, and reduced eGFR could not be confirmed as persistent for ≥3 months; therefore, the incident CKD outcome in this study should be interpreted as a study‐defined outcome rather than clinically confirmed CKD. Because kidney outcomes were assessed only at scheduled follow‐up visits, the exact timing of reduced kidney function onset could not be determined, and some degree of interval censoring may have been present. eGFR was estimated using serum creatinine rather than cystatin C, which may be influenced by non‐GFR determinants. In addition, kidney function decline was assessed using the annual eGFR slope over a relatively long follow‐up period between FU2 and FU3, which primarily reflects chronic changes in kidney function. Due to the spacing of measurements, we were unable to distinguish acute from chronic changes in eGFR. Therefore, our findings should be interpreted in the context of long‐term kidney function decline rather than short‐term fluctuations. Finally, participants with reduced kidney function (eGFR <60 mL/min/1.73 m^2^) were excluded to focus on incident CKD and kidney function decline among individuals with preserved baseline kidney function. As a result, our findings may not be directly generalizable to individuals with pre‐existing CKD or more advanced kidney impairment, in whom the associations between blood pressure patterns and kidney outcomes may differ. Excluded participants generally had a less favorable cardiovascular risk profile than included participants, and some degree of selection bias related to participant exclusion and complete‐case analysis cannot be excluded.

## 5 Conclusion

In this prospective cohort, higher cumulative BP exposure, particularly cumulative SBP, and greater visit‐to‐visit BP variability were associated with a faster decline in kidney function. These findings highlight the predominant role of long‐term BP burden in kidney deterioration and underscore the importance of considering BP patterns over time in CKD prevention.

## Author Contributions

**Sisi Xie**: investigation, methodology, formal analysis, visualization, writing – original draft. **Peter Vollenweider** and **Julien Vaucher**: review and editing. **Pedro Marques‐Vidal**: conceptualization, methodology, resources, data curation, writing‐supervision. Pedro Marques‐Vidal had full access to the data and is the guarantor of the study.

## Funding

The CoLaus|PsyCoLaus study was supported by research grants from GlaxoSmithKline, the Faculty of Biology and Medicine of Lausanne, the Swiss National Science Foundation (grants 33CSCO‐122661, 33CS30‐139468, 33CS30‐148401, 33CS30_177535, 31003A‐182420, 324730_204523 and 320030_220190) and the Swiss Personalized Health Network (grant 2018DRI01). ToxiLaus was supported by a research grant from the Fondation pour la recherche sur le diabète (https://fondation‐diabete.ch). The funders had no role in the design of the study; in the collection, analyses, or interpretation of data; in the writing of the manuscript; or in the decision to publish the results.

## Ethics Statement

The CoLaus|PsyCoLaus study was approved by the Ethics Commission of the Canton of Vaud (PB_2018‐00038; reference 239/09).

## Consent

Written informed consent was obtained from all participants.

## Conflicts of Interest

The authors declare no conflicts of interest.

## Supporting information




**Supplementary Information**: Supplemental Material.docx

## Data Availability

The data of the CoLaus|PsyCoLaus study used in this article cannot be fully shared as they contain potentially sensitive personal information on participants. According to the Ethics Committee for Research of the Canton of Vaud, sharing these data would be a violation of the Swiss legislation with respect to privacy protection. However, coded individual‐level data that do not allow researchers to identify participants are available upon request to researchers who meet the criteria for data sharing of the CoLaus|PsyCoLaus Datacenter (CHUV, Lausanne, Switzerland). Any researcher affiliated to a public or private research institution who complies with the CoLaus|PsyCoLaus standards can submit a research application to research.colaus@chuv.ch or research.psycolaus@chuv.ch. Proposals requiring baseline data only will be evaluated by the baseline (local) Scientific Committee (SC) of the CoLaus and PsyCoLaus studies. Proposals requiring follow‐up data will be evaluated by the follow‐up (multicentric) SC of the CoLaus|PsyCoLaus cohort study. Detailed instructions for gaining access to the CoLaus|PsyCoLaus data used in this study are available at www.colaus‐psycolaus.ch/professionals/how‐to‐collaborate/. Kindly
