# Supplementary material for: Associations of Long‐Term Blood Pressure Burden and Variability With Kidney Function Decline
Source: J Clin Hypertens (Greenwich). 2026 Jun 1;28(6):e70306. doi: 10.1111/jch.70306 (PMC13239535; doi:10.1111/jch.70306)
Supplement: Supplementary file 1 — Supplementary Information: Supplemental Material.docx [file JCH-28-e70306-s001.docx]

**Supplementary materials:**

**Table S1.** Means and standard deviations of BP exposure metrics included in the analyses.

| **BP metric** | **Unit** | **Mean** | **SD** |
| --- | --- | --- | --- |
| SBPcum | mmHg·year | 1337.4 | 163.6 |
| SBPtwa | mmHg | 124.0 | 14.5 |
| SBP_SD | mmHg | 8.2 | 5.5 |
| SBP_CV | % | 6.5 | 4.0 |
| SBP_VIM | unitless | 8.4 | 5.0 |
| DBPcum | mmHg·year | 837.3 | 101.6 |
| DBPtwa | mmHg | 77.7 | 8.9 |
| DBP_SD | mmHg | 5.9 | 3.7 |
| DBP_CV | % | 7.6 | 4.5 |
| DBP_VIM | unitless | 6.0 | 3.5 |

**Table S2.** Characteristics of included and excluded participants at study baseline.

| **Variables** | **Included participants (n=2821)** | **Excluded due to missing covariates (n=442)** | **Excluded due to missing FU2 or FU3 or BP data (n=3170)** | ***p*-value** |
| --- | --- | --- | --- | --- |
| Age, years | 49.8 ± 9.4 | 51.2 ± 10.4 | 54.3 ± 11.1 | **<0.001** |
| Female sex, % | 1555 (55.1) | 232 (52.5) | 1581 (49.9) | **<0.001** |
| Education level, % |  |  |  | **<0.001** |
| Low | 1309 (46.4) | 287 (65.2) | 2007 (63.5) |  |
| Middle | 819 (29.0) | 86 (19.6) | 645 (20.4) |  |
| High | 693 (24.6) | 67 (15.2) | 507 (16.1) |  |
| Smoking status, % |  |  |  | **<0.001** |
| Never | 1220 (43.3) | 161 (36.5) | 1222 (38.6) |  |
| Former | 940 (33.3) | 155 (35.2) | 970 (30.7) |  |
| Current | 661 (23.4) | 125 (28.3) | 973 (30.7) |  |
| Alcohol consumption(unit), % |  |  |  | **<0.001** |
| None | 708 (25.1) | 112 (25.3) | 1010 (31.9) |  |
| 1-13/week | 1717 (60.9) | 250 (56.6) | 1525 (48.1) |  |
| 14-27/week | 320 (11.3) | 63 (14.2) | 487 (15.3) |  |
| 28+/week | 76 (2.7) | 17 (3.9) | 148 (4.7) |  |
| BMI, kg/m² | 25.0 ± 4.1 | 25.9 ± 4.2 | 26.4 ± 4.8 | **<0.001** |
| SBP, mmHg | 124.3 ± 15.8 | 126.5 ± 17.1 | 130.4 ± 18.6 | **<0.001** |
| DBP, mmHg | 78.1 ± 10.5 | 78.9 ± 10.8 | 80.3 ± 11.1 | **<0.001** |
| Diabetes, % | 73 (2.6) | 26 (5.9) | 292 (9.2) | **<0.001** |
| LDL cholesterol, mmol/L | 3.3 ± 0.9 | 3.3 ± 0.9 | 3.3 ± 1.0 | 0.259 |
| Antihypertensive medication, % | 333 (11.8) | 74 (16.7) | 767 (24.2) | **<0.001** |
| Baseline eGFR, mL/min/1.73m^2^ | 91.1 ± 13.7 | 92.1 ± 14.4 | 89.7 ± 15.6 | **<0.001** |

Results are presented as mean ± SD for continuous variables and number (column percentage) for categorical variables. P-values were obtained using one-way analysis of variance (ANOVA) for continuous variables and chi-square tests for categorical variables. Participants with prevalent CKD at follow-up 2 (n=300) were excluded from this comparison because they were excluded by study design.

**Table S3.** Characteristics of participants at follow-up 2 according to inclusion status in the analytical sample.

| **Variables** | **Included participants (n=2821)** | **Excluded due to missing covariates (n=442)** | **Excluded due to missing FU3 or BP data (n=1318)** | ***p*-value** |
| --- | --- | --- | --- | --- |
| Age, years | 60.6 ± 9.3 | 62.1 ± 10.4 | 65.5 ± 10.8 | **<0.001** |
| Female sex, % | 1555 (55.1) | 232 (52.5) | 725 (55.0) | 0.579 |
| Education level, % |  |  |  | **<0.001** |
| Low | 1309 (46.4) | 287 (65.2) | 821 (62.3) |  |
| Middle | 819 (29.0) | 86 (19.5) | 278 (21.1) |  |
| High | 693 (24.6) | 67 (15.2) | 218 (16.6) |  |
| Smoking status, % |  |  |  | **0.006** |
| Never | 1212 (43.0) | 104 (38.2) | 452 (40.1) |  |
| Former | 1104 (39.1) | 105 (38.6) | 419 (37.2) |  |
| Current | 505 (17.9) | 63 (23.2) | 255 (22.7) |  |
| Alcohol consumption(unit), % |  |  |  | **<0.001** |
| None | 659 (23.4) | 55 (37.4) | 370 (36.5) |  |
| 1-13/week | 1771 (62.8) | 71 (48.3) | 508 (50.1) |  |
| 14-27/week | 321 (11.4) | 18 (12.2) | 107 (10.6) |  |
| 28+/week | 70 (2.5) | 3 (2.0) | 28 (2.8) |  |
| BMI, kg/m² | 26.0 ± 4.5 | 27.0 ± 4.7 | 26.9 ± 5.0 | **<0.001** |
| SBP, mmHg | 124.7 ± 16.6 | 128.4 ± 18.8 | 130.3 ± 19.4 | **<0.001** |
| DBP, mmHg | 77.1 ± 10.2 | 78.6 ± 11.1 | 78.6 ± 11.4 | **<0.001** |
| Diabetes, % | 192 (6.8) | 55 (14.2) | 180 (17.3) | **<0.001** |
| LDL cholesterol, mmol/L | 3.2 ± 0.9 | 3.2 ± 1.0 | 3.1 ± 1.0 | **<0.001** |
| Antihypertensive medication, % | 750 (26.6) | 162 (36.7) | 565 (42.9) | **<0.001** |
| FU2 eGFR, mL/min/1.73m^2^ | 87.3 ± 12.5 | 88.2 ± 12.8 | 86.8 ± 12.7 | 0.177 |

Results are presented as mean ± SD for continuous variables and number (column percentage) for categorical variables. P-values were obtained using one-way analysis of variance (ANOVA) for continuous variables and chi-square tests for categorical variables. Participants with prevalent CKD at follow-up 2 (n=300) and those without follow-up 2 attendance (n=1852) were excluded from this comparison because follow-up 2 characteristics were unavailable or participants were excluded by study design. This table was generated to assess potential selection bias related to participant exclusion and missing data.

**Table S4.** Sensitivity analysis: associations of blood pressure metrics (per 1-SD increase) with the annual percent change in eGFR between follow-up 2 and follow-up 3 across three adjustment models.

| **BP metric**  **(Per 1-SD)** | **Model 1**  **β (95%CI)** | ***p*-value** | **Model 2**  **β (95%CI)** | ***p*-value** | **Model 3**  **β (95%CI)** | ***p*-value** | **FDR (q)** |
| --- | --- | --- | --- | --- | --- | --- | --- |
| SBPcum | -0.22 (-0.35, -0.09) | **0.001** | -0.22 (-0.35, -0.09) | **0.001** | -0.19 (-0.32, -0.07) | **0.002** | **0.005** |
| SBPtwa | -0.23 (-0.36, -0.09) | **0.001** | -0.22 (-0.35, -0.09) | **0.001** | -0.20 (-0.32, -0.07) | **0.002** | **0.005** |
| SBP_SD | -0.18 (-0.29, -0.07) | **0.001** | -0.18 (-0.29, -0.07) | **0.001** | -0.18 (-0.29, -0.08) | **0.001** | **0.005** |
| SBP_CV | -0.16 (-0.26, -0.05) | **0.004** | -0.16 (-0.27, -0.05) | **0.004** | -0.16 (-0.27, -0.06) | **0.002** | **0.005** |
| SBP_VIM | -0.12 (-0.23, -0.02) | **0.023** | -0.12 (-0.23, -0.02) | **0.021** | -0.13 (-0.23, -0.03) | **0.011** | **0.014** |
| DBPcum | -0.15 (-0.26, -0.03) | **0.013** | -0.14 (-0.26, -0.02) | **0.017** | -0.14 (-0.25, -0.03) | **0.016** | **0.018** |
| DBPtwa | -0.14 (-0.26, -0.02) | **0.018** | -0.13 (-0.25, -0.01) | **0.029** | -0.13 (-0.24, -0.02) | **0.023** | **0.023** |
| DBP_SD | -0.17 (-0.28, -0.06) | **0.002** | -0.17 (-0.28, -0.06) | **0.002** | -0.17 (-0.27, -0.07) | **0.001** | **0.005** |
| DBP_CV | -0.16 (-0.26, -0.05) | **0.004** | -0.16 (-0.26, -0.05) | **0.004** | -0.16 (-0.26, -0.05) | **0.003** | **0.005** |
| DBP_VIM | -0.15 (-0.26, -0.05) | **0.004** | -0.16 (-0.26, -0.05) | **0.004** | -0.15 (-0.26, -0.05) | **0.003** | **0.005** |

Results are expressed as adjusted β coefficients and 95% confidence intervals (CIs) derived from multivariable linear regression models. Blood pressure metrics were standardized (per 1-SD increase). The outcome was the annual percent change in eGFR between FU2 and FU3. Model 1 was adjusted for age, sex, education, smoking status, alcohol consumption, body mass index, diabetes, LDL cholesterol, and antihypertensive medication use at FU2. Model 2 included all covariates in Model 1 with additional adjustment for baseline eGFR. Model 3 included all covariates in Model 1 with additional adjustment for eGFR at FU2. FDR-adjusted q values were calculated using the Benjamini–Hochberg method. ***Abbreviations:*** SBP, systolic blood pressure; DBP, diastolic blood pressure; cum, cumulative exposure; twa, time-weighted average; SD, standard deviation; CV, coefficient of variation; VIM, variability independent of the mean; eGFR, estimated glomerular filtration rate.

**Figure S1.** Flowchart of participant selection.

Participants were excluded sequentially based on predefined criteria.

**Figure S2.** Subgroup analyses of the association between cumulative systolic blood pressure and annual eGFR slope.

Subgroup analyses evaluating the association between cumulative systolic blood pressure (per 1-SD increase) and annual eGFR slope. Estimates were adjusted for age, sex, education level, smoking status, alcohol consumption, body mass index, diabetes, hypertension status, LDL cholesterol, and antihypertensive medication use at FU2 (except when the variable was the stratifying factor). Data points represent adjusted β coefficients with 95% confidence intervals. P-values for interaction are shown for each subgroup.
